# Evaluating the Integration of Mindfulness in School Settings: Evidence, Limitations, and Contextual Adaptations

**DOI:** 10.1192/j.eurpsy.2026.10783

**Published:** 2026-07-31

**Authors:** B. Çam

**Affiliations:** Institute of Psychiatry, Psychology and Neuroscience, King’s College London, London, United Kingdom

## Abstract

**Introduction:**

Mindfulness, defined as present-focused, non-judgmental awareness, has gained attention in schools for its potential to reduce stress, enhance attention, and support emotional resilience in students (Kabat-Zinn, 1994).

**Objectives:**

This study critically reviews empirical evidence on school-based mindfulness programs, assessing their effectiveness in improving students’ emotional regulation, cognition, and well-being, while considering contextual and ethical challenges of implementation.

**Methods:**

A comprehensive review of systematic reviews and meta-analyses published between 2014 and 2023 was conducted, focusing on mindfulness-based interventions across educational levels from preschool to university. Key studies included Zenner et al. (2014), Zoogman et al. (2015), Mettler et al. (2023), and Volanen et al. (2016). Moderator analyses, methodological quality, effect sizes, and contextual factors such as facilitator expertise, developmental stage, and intervention design were examined.

**Results:**

Mindfulness-based programs showed small to moderate positive effects on attention, cognitive performance, emotional resilience, and school adjustment, with stronger outcomes in clinical populations and older adolescents. Program efficacy was influenced by facilitator expertise, intervention design, and contextual alignment. Effects on interpersonal skills and teacher-reported behavior were minimal. Methodological heterogeneity, short follow-ups, and limited school-context adaptation reduced generalizability. Ethical considerations regarding cultural sensitivity and pathologizing systemic issues were noted.

**Conclusions:**

Mindfulness shows promise as a school-based intervention when programs are well-designed, context-sensitive, and targeted to students most likely to benefit. Evidence supports improvements in emotional regulation and well-being, but mindfulness should not be applied universally. Future research should focus on personalized, developmentally appropriate approaches integrated into broader systemic strategies. Careful and critical implementation remains essential.

**Disclosure of Interest:**

None Declared

